# Mega Clonality in an Aquatic Plant—A Potential Survival Strategy in a Changing Environment

**DOI:** 10.3389/fpls.2018.00435

**Published:** 2018-04-06

**Authors:** Eric Bricker, Ainsley Calladine, Robert Virnstein, Michelle Waycott

**Affiliations:** ^1^Department of Environmental Sciences, University of Virginia, Charlottesville, VA, United States; ^2^Department for Environment and Water, State Herbarium of South Australia, Adelaide, SA, Australia; ^3^School of Biological Sciences, The University of Adelaide, Adelaide, SA, Australia; ^4^Seagrass Ecosystems Analysts, East Palatka, FL, United States

**Keywords:** marine angiosperm, seagrass, aquatic plant, clonality, microsatellites, adaptive mechanisms, windows of opportunity

## Abstract

Many ecosystems are experiencing rapid transformations due to global environmental change. Understanding how ecological shifts affect species persistence is critical to modern management strategies. The edge of a species range is often where physiological tolerances are in conflict with ability to persist. Extreme examples of clonality over large spatial and temporal scales can occur where the life history of a species allows for it. We examine extreme clonality in an aquatic plant species at the edge if its range. Here we describe an ancient seagrass clone of unprecedented size inhabiting a 47 km stretch of a central Florida estuary, the Indian River Lagoon (IRL). Amongst the largest clones on earth detected, this *Thalassia testudinum* (turtlegrass) genet had ramets dispersed across 47 km of this water body. Indeed among 382 samples collections along the length of the IRL, 89% were a single shared multilocus genotype. Furthermore, this clone was the only genet detected at 63% of sample sites. The presence of such a large clone demonstrates they can form and persist over long periods. In addition, we must challenge the paradigm that fragmentation is not possible in this species. Reliance on clonality is an expected component of a classic ‘bet-hedging’ strategy enabling persistence on timescales typically not considered, including millennia. At locations near ocean inlets we did find a few other individuals of *T. testudinum* supporting the concept that recruitment is dispersal limited. These additional clones indicate there is the potential, albeit limited, for seeds based recruitment to occur when environmental conditions are favorable during a “window of opportunity.” Extreme clonality represents a potential strategy for survival such that in the extreme, clonal populations of a species would be the first to decline or disappear if conditions extend beyond the adaptability of the local genotype. This disappearance possibility makes the species a potential sentinel of system decline.

## Introduction

Giant, long-lived clones have been documented in diverse organismal groups and numerous times among seagrasses (Arnaud-Haond et al., 2012). Enormous clonal individuals may be 1000s, 10s, or even 100s of 1000s of years old (de Witte and Stocklin, 2010; Arnaud-Haond et al., 2012). Longevity allows persistence of genets through periods of poor or non-existent sexual reproduction (Eriksson and Froborg, 1996), a life history strategy that stabilizes populations amidst stochastic perturbations (de Witte and Stocklin, 2010). However, persistence relying on asexual reproduction may limit reproductive success for sexually derived offspring, especially if clones occupy the niche space of potential recruits. Environmental stress and niche occupancy could potentially lead to a population comprised of a single individual (genet), something rarely seen in natural systems (Ellstrand and Roose, 1987). Mono-genet (uniclonal) populations in peripheral populations conform to the central marginal hypothesis (Petit et al., 2003). Though not axiomatic (Eckert et al., 2008), the central marginal hypothesis predicts genetic diversity will be lowest at the species margin because biological tolerances of a species reach their limit, a prediction that is observed rarely across diverse taxonomic groups (Petit et al., 2003; Eckert et al., 2008). Usually, for species with long life histories, assessing the success of sexual reproduction will be inferential due to the temporal scales associated with recruitment processes and limited observational and sampling efforts that will be possible.

Seagrasses are ecologically successful marine angiosperms, particularly in terms of their dominance across broad niche occupancy and geographic extent (Orth et al., 2006). They have a long evolutionary history and belong to ancient aquatic monocotyledon lineages (Les et al., 1997). Seagrasses exist as clones that are derived from vegetative growth and fragment isolation due to rhizome decay yet remain capable of sexual reproduction via seed (Les, 1988; Waycott, 1995, 1998; Reusch, 2001; Olsen et al., 2004; Alberto et al., 2006; Reusch, 2006; Waycott et al., 2006; Hughes and Stachowicz, 2009; van Dijk and van Tussenbroek, 2010; Kendrick et al., 2012; McMahon et al., 2014).

Variability in type and level of disturbance leads to differing reliance on sexual and asexual propagation and potentially a bet-hedging strategy (*sensu* Seger and Brockmann, 1987). Stochasticity in the success of sexual recruitment makes vegetative endurance vital to the persistence of populations of these organisms. The ongoing study of bet-hedging strategies has advanced our theoretical understanding of this evolutionary strategy (Philippi and Seger, 1989), however, there remain few examples of this life-history strategy in natural systems.

*Thalassia testudinum*, is a seagrass known as turtlegrass, a favored food of marine turtles. Turtlegrass is a late-successional species that dominates throughout the Caribbean, Gulf of Mexico and into the Atlantic (den Hartog, 1970). This vast geographic distribution exposes turtlegrass to a wide variety of ecological and environmental conditions. The species yields enormous biomass and is efficient at resource translocation indicating a strong reliance on vegetative reproduction, and large dispersed clones do exist within populations (van Dijk and van Tussenbroek, 2010). Despite these characteristics exemplifying the extent and importance of vegetative growth, populations have been found to be genetically diverse (van Dijk et al., 2009; Bricker et al., 2011) suggesting recruitment from seed has a strong influence on population structure. Genetic diversity has been found to be high in Florida Bay (Bricker et al., 2011) where density of benthic coverage (Zieman et al., 1989) means trillions of turtlegrass ramets make it one of the largest seagrass populations in the world. We investigated whether turtlegrass maintained high genetic diversity at the species margin in a system that experiences temperature stress (i.e., too cold) regularly. We suggest that it is likely that turtlegrass hedges its bets at the species margin in a remarkably different way than observed in other populations (Bricker et al., 2011).

## Materials and Methods

**FIGURE 1:**
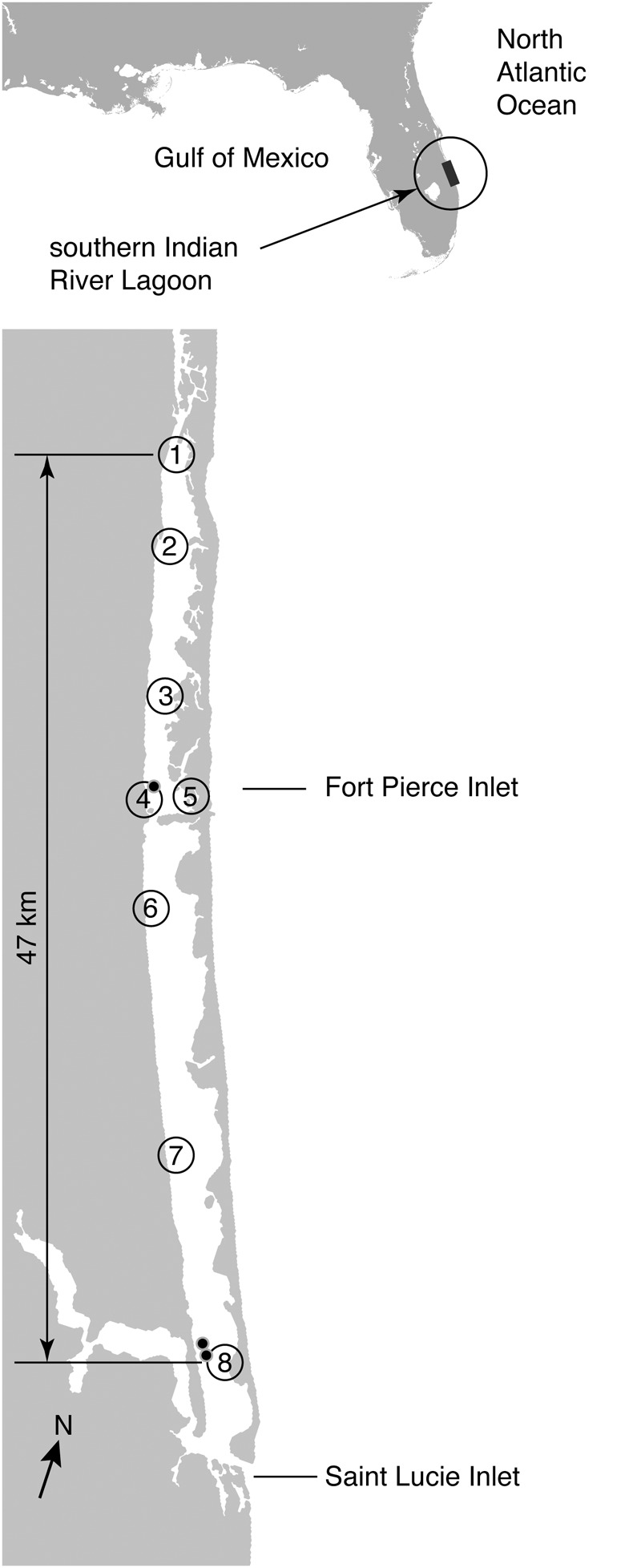
Distribution of samples of *Thalassia testudinum* taken across the Indian River Lagoon (IRL). The numbers indicate the spatial location of eight sampling locations for *Thalassia testudinum* genotypes. Black dots indicate sampling sites where more than one multi-locus genotype (clone) was detected. Note there are two transects at site 8 and these are visualized by two location markers on the map.

Fresh tissues samples from known spatial coordinates were collected from eight sites in the IRL (**Figure 1**). The IRL provided an ecosystem which was able to be compared to Florida Bay (FB) as it represented a lagoon-type estuary with limited communication with surrounding marine systems. The Indian River Lagoon (IRL) also represents the most northern extent of *Thalassia testudinum* along the east coast of North America (van Tussenbroek et al., 2010). Considerable data was already available on the genetic structure of *Thalassia testudinum* populations in Florida Bay using the same marker systems enabling data comparison (Bricker et al., 2011). Sampling in the IRL for this study was synoptic, the sites selected based on recent seagrass survey (Morris and Virnstein, 2004). The synoptic survey harvested samples from 5 sites where *Thalassia* had been recorded to have occurred previously (Morris and Virnstein, 2004). Sampling was also taken at different times, first five of the northern locations in 2002 (the synoptic survey), was then expanded to a wider range once preliminary results were obtained. This enabled the study to expand to include the southern sites which were sampled between 2011 and 2015. In all we have sampled from 8 sites genotyping 382 samples. The northern limit of sampling occurred at 27.586875°, -80.364159° and the southern limit was 27.189788°, -80.189605° (**Figure 1**).

Fresh tissues samples, in the form of short shoots including the sheathed shoot meristem, were taken from known spatial coordinates (**Figure 1**). At each site samples were collected a minimum of 5 m apart from each other at known locations. In some cases the ramet density was too low to collect at 5 m intervals, in these cases 5 m was the minimum collection interval some samples were collected further apart. At most of the collection sites there were two parallel transects sampled across the area. The goal at each site was to harvest approximately 30 ramets per site, however, between low ramet density, and lab error that eliminated some samples the number of samples that ended up being analyzed varied site to site (Supplementary Table 1). Samples were transported to a University of Virginia laboratory where DNA extractions and PCR reactions were undertaken. Analysis genuinely followed the protocol in Bricker et al. (2011).

All samples were genotyped using co-dominant genetic markers previously published van Dijk et al. (2007). PCR products, based on direct, single locus based, amplification with primers containing fluorescent tags, were analyzed by capillary electrophoresis on a MegaBACE 1000^TM^ (GE Biosciences) with an internal ET-ROX 400^TM^ size standard (GE Biosciences). Up to 14 loci per individual sample were amplified, of which only 8 were polymorphic among the IRL samples, perhaps to be expected in this edge of range location. Alleles for each locus were scored from chromatographic traces using Genetic Profiler Suite v2.2. Genotype and sibling probability was estimated using GenClone (Arnaud-Haond and Belkhir, 2007). Codominant marker based genetic distance (GD, genetic distance binary, in GenAlEx v6.5, the count of whole number 1-(shared number of alleles)), number of effective alleles, observed and expected heterozygosity along with a distance standardized, Principal Coordinates Analysis (PCoA) and pairwise genetic distance based on Fst and AMOVA to estimate partitioning of genetic diversity were computed using GenAlEx v6.5 (Peakall and Smouse, 2012).

## Results

A total of 382 *Thalassia testudinum* ramets collected from 8 sites across a 47 km transect of the IRL (**Figure 1**). Genetic analysis showed that 285 or 89% of the total sample were a single shared multilocus genotype (MLG or clone, **Table 1** and Supplementary Table 1). Both the genotype identity (P_gen_) and sibling probability (P_sex_) (*p* < 0.001) statistically substantiated clonality. These measures allow confidence that the genotype data is most likely due to clonality rather than two individuals which are either highly similar due to being related (e.g., siblings) or very low diversity (i.e., no heterozygosity), neither option is observed here. This single genet (*Tt*-IRL1) was the only individual detected at 5 of the 8 sampling sites and present at all of the sampling sites. In addition and further confirming clonality, *Tt*-IRL1 possessed 7 fixed heterozygous loci (**Table 1**) of the 8 known to be polymorphic in adjacent populations (Bricker et al., 2011). The other genets detected were collected near Ft. Pierce at site 4, Commercial Boat Dock, (3 additional genotypes) and St. Lucie inlets (22 additional genets) near the southern end of the IRL, (**Figure 1**–sites 4 and 8, **Table 2** and Supplementary Table 2). Among the additional 25 genets detected in this study many were closely related (e.g., Supplementary Table 2), none were closely related to *Tt*-IRL1.

**Table 1:** Diploid multilocus genotype fragment lengths of scored alleles detected across all samples designated to be clone *Tt*-IRL1.

	Th1MS	TTMS-GA6	TTMS-GA8	TTMS-GA12	TTMS-TCT58	TTMS-GGT59	TTMS-GA72	TTMS-GT104
Allele 1	**156**	**126**	**236**	**155**	**179**	237	**225**	188
Allele 2	**158**	**130**	**260**	**173**	**191**	237	**229**	188

*Fixed heterozygotes are indicated in **bold**. Total number of ramets sharing this multi-locus match *n* = 285 found in locations up to 47 km apart. Locus names correspond to those published in the primer note for comparative purposes (van Dijk et al., 2007).*

**Table 2:** Diploid multilocus genotype fragment lengths of scored alleles detected across all samples in addition to clone *Tt*-IRL1 (listed at 0^∗^ at bottom of table for comparative purposes).

	Region in IRL	N	Th1MS		TTMS-GA6		TTMS-GA8		TTMS-GA12		TTMS-TCT58		TTMS-GGT59		TTMS-GA72		TTMS-GT104	
1	IRL-SL	2	150	154	124	128	218	236	161	173	167	179	237	237	225	227	188	188
2	IRL-SL	2	150	154	124	128	222	236	173	173	167	179	237	237	227	231	188	188
3	IRL-SL	3	150	154	124	128	236	236	161	173	167	179	237	237	225	227	186	188
4	IRL-SL	3	150	154	124	128	236	236	161	173	167	179	237	237	225	227	188	188
5	IRL-SL	2	150	154	124	128	236	236	173	173	167	179	237	237	227	231	186	188
6	IRL-SL	3	150	156	124	124	236	236	173	177	167	179	237	237	225	233	188	192
7	IRL-SL	2	150	156	124	128	236	236	173	173	167	179	237	237	225	233	188	192
8	IRL-SL	2	150	156	124	128	236	236	173	177	167	179	237	237	225	233	188	192
9	IRL-SL	2	154	156	124	128	236	236	173	173	176	179	228	237	227	233	188	192
10	IRL-SL	1	146	156	124	128	236	236	171	173	167	179	237	237	231	233	188	192
11	IRL-SL	1	148	154	124	128	236	236	161	173	167	179	237	237	225	227	192	192
12	IRL-SL	1	150	154	124	128	220	236	173	173	167	179	237	237	227	231	186	188
13	IRL-SL	1	150	154	124	128	226	236	161	173	167	179	237	237	225	227	188	188
14	IRL-SL	1	150	154	124	128	236	236	161	173	167	179	237	237	225	233	188	192
15	IRL-SL	1	150	154	124	128	236	236	173	173	167	179	237	237	227	231	188	188
16	IRL-SL	1	150	154	124	128	236	236	173	177	167	179	237	237	227	231	188	188
17	IRL-SL	1	152	154	124	128	222	236	161	173	167	179	237	237	225	227	190	192
18	IRL-SL	1	154	154	124	128	236	236	161	173	167	179	237	237	225	227	192	192
19	IRL-SL	1	154	154	124	128	236	236	173	177	167	179	237	237	225	233	192	192
20	IRL-SL	1	154	156	124	128	236	236	171	173	167	179	237	237	227	233	188	192
21	IRL-SL	1	154	156	124	128	236	236	171	173	176	179	237	237	227	233	188	192
22	IRL-SL	1	156	156	124	128	236	236	173	173	167	179	237	237	231	233	188	192
23	IRL-FP	1	156	158	126	130	236	260	155	172	179	191	237	237	225	229	188	188
24	IRL-FP	1	156	158	126	130	236	260	170	172	179	179	237	237	229	233	188	188
25	IRL-FP	1	156	158	126	130	236	260	172	174	179	191	237	237	227	227	188	192
0^∗^	IRL Big Clone	339	156	158	126	130	236	260	155	173	179	191	237	237	225	229	188	188

*Locus names correspond to those published in the primer note for comparative purposes (van Dijk et al., 2007).*

**FIGURE 2:**
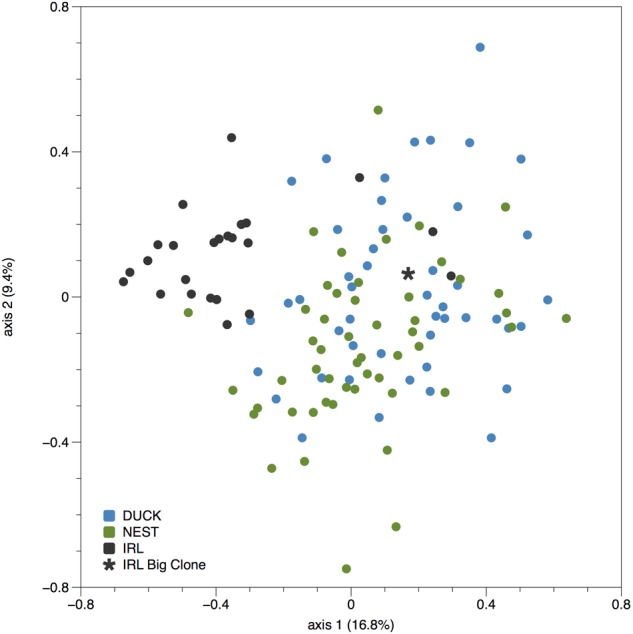
Principal coordinates analysis of genetic distance (standardized) among individual *Thalassia testudinum* samples analyzed from two locations in Florida Bay (Duck and Nest keys, Bricker et al., 2011) and the Indian River Lagoon (IRL). Note the sample indicated by a ^∗^ is the Mega-clone sample.

Comparison of the IRL genetic diversity with the two closest populations in Florida Bay, Duck Key and Nest Key, see Bricker et al. (2011) for comparative data, exhibited limited genotypic similarity (**Table 3**). Axis 1 and 2 ordination plot revealed the majority of IRL samples clustered to one side of the Duck Key and Nest Key samples, more than 200 km distant (**Figure 2**). Interestingly, the IRL Mega-clone was found within the broader cluster of DUCK and NEST samples in the ordination space (**Figure 2**). The pairwise genetic distance (Fst based) among the three locations was very low between the two Florida Bay populations (DUCK and NEST, Fst = 0.024) and considerably higher between the IRL and both DUCK and NEST (Fst = 0.14; 0.13 respectively). AMOVA results established 9% of the variance is partitioned among the three populations, the remainder within populations.

**Table 3:** Comparison of population genetic measures of diversity across all Indian River Lagoon samples, and two population samples closest to the IRL in Florida Bay (Duck and Nest keys). Estimates^a^ calculated in GenAlEx 6.5 (Peakall and Smouse, 2012).

Pop		*N*	Na	Ne	*I*	Ho	He	uHe	*F*
DUCK	Mean	52.000	7.000	2.962	1.265	0.577	0.603	0.609	0.027
	*SE*	0.000	0.779	0.436	0.153	0.059	0.065	0.065	0.060
NEST	Mean	54.000	6.250	2.561	1.116	0.535	0.541	0.547	–0.005
	*SE*	0.000	0.921	0.364	0.163	0.070	0.074	0.075	0.045
IRL	Mean	38.000	5.000	2.386	0.972	0.678	0.506	0.513	–0.288
	*SE*	0.000	0.681	0.336	0.155	0.123	0.083	0.084	0.085
Total	Mean	48.000	6.083	2.636	1.118	0.596	0.550	0.556	–0.089
	*SE*	1.484	0.474	0.216	0.090	0.050	0.042	0.042	0.047

*^*a*^Metrics generated by GenAlEx v6.5 (Peakall and Smouse, 2012). Na, No. of Different Alleles; Ne, No. of Effective Alleles = 1/(Sum pi^*2*^); I, Shannon’s Information Index = -1^∗^ Sum [pi ^∗^ Ln (pi)]; Ho, Observed Heterozygosity = No. of Hets/N; He, Expected Heterozygosity = 1 - Sum pi^*2*^; uHe, Unbiased Expected Heterozygosity = (2N/(2N-1)) ^∗^ He; F, Fixation Index = (He - Ho)/He = 1 - (Ho/He).*

## Discussion

To date no individual plant genet (clone) has been detected covering a larger area (Arnaud-Haond et al., 2012). If we infer a continual growth model, the size of this particular genet would signify finding the oldest living thing on earth. However, we do not subscribe to this model of growth to explain the pattern of genets in the south-eastern United States IRL population. These results, in fact, represent evidence that recruitment via fragmentation is possible in this seagrass species. We suggest there are alternative explanations for this mega-clone are other than the possibility that it grew to this size. The proto-clone may have been fragmented into vegetative sub-units that survived hydrological or wind driven transport to inhabit areas across the IRL (McMahon et al., 2014). It is possible that this clone is a highly adapted genotype that out competed other genotypes in the edge of range location. This final option, we suggest is unlikely as there are in fact other, unrelated genotypes in the region (**Figure 1**). These other clones are only present adjacent to the entry points to the IRL suggesting dispersal limitation rather than selection. However, without testing the different survivorship traits of the various genets in the IRL, we cannot completely dismiss this as a possibility.

We dismiss the possibility *Thalassia testudinum* grew linearly to occupy this system as published maximum growth rates for turtlegrass, 19-35 cm/year; (van Tussenbroek et al., 2006), would lead to an estimate of the age range for the mega-clone between 120,000 and 220,000 years old. This value would make this clone the oldest living thing on earth (de Witte and Stocklin, 2010; Arnaud-Haond et al., 2012). However, the coastal geomorphological structure of the IRL is at best around 6000 years old (White and Florida Bureau of Geology, 1970) and suitable habitat for seagrass growth may be as little as 2000 years old. In addition, what is known about the coastal paleo-history of the east coast of Florida (White and Florida Bureau of Geology, 1970) reveals no suitable habitat for the mega-clone allowing it to have grown with sea-level rise into the newly formed IRL. Additionally, the rate of sea level rise during recent history has been greater than the published growth rates for turtlegrass. Thus, we dismiss direct growth as a possible scenario.

If the clone did not grow to this size, then dispersal of viable vegetative fragments is the only model that describes the current range of this genet. Experiments on *Thalassia hemprichii*, the Indo-West Pacific sister species to *Thalassia testudinum*, demonstrate the potential for fragments to survive enabling fragment based recruitment (Wu et al., 2016). Numerous processes could dislodge belowground roots and rhizomes: grazing by manatees; human uprooting from dredging, boat propellers or anchors; and natural uprooting from wave action during storms. While the exact mechanism remains obscure, we suggest that in most circumstances fragments resulting from grazing animals such as manatees would be too small to remain viable. During field work conducted by the authors, fragments have been observed *in situ*. The IRL has a long history of substantial disturbance from boating traffic, dredging, and other types of urban development (Crawford et al., 1997). We recognize that survival of vegetative fragments and their recruitment into new habitat may be rare, an inference made due to turtlegrass not having fared well in experimental transplant studies (Fonseca et al., 1996). Rare convergence of numerous factors would be required for this recruitment strategy to operate successfully.

Clonal expansion via movement of fragments forming a long-lived seagrass suggests a dispersal mechanism that contrasts with other examples of ancient seagrass clones (Arnaud-Haond et al., 2012). This finding also conflicts with the commonly held understanding that seagrasses do not colonize via fragmentation (den Hartog, 1970). However, an increasing number of studies are documenting the possibility and providing direct and indirect evidence that fragments of plants may in fact colonize (Kenworthy et al., 2002; Campbell, 2003; Di Carlo et al., 2004; Hall et al., 2006; Sherman et al., 2016; Wu et al., 2016). In addition, by adopting a fragmentation model to explain the current distribution makes it difficult to precisely estimate the age of the mega-clone *Tt*-IRL1. However, even under optimistic models of fragmentation frequency, the extended presence of the mega-clone *Tt*-IRL1 suggests it would be very old.

Our detection of a widespread, mono-clonal, long lived individual of turtlegrass poses a conundrum for survival of this species as reduction in population size to a single clone of one sex is a risky persistence strategy. Primarily, this is due to turtlegrass being dioecious (having separate male and female plants). As a result essentially this genet cannot reproduce sexually because mega-clone *Tt*-IRL1 has no mate throughout almost all of its’ range. Longevity as a method of bet-hedging ensures survival by continuing to be present in the population until a time when sexual offspring may be produced. However, the requirement for a mate means that the opportunity to mate for this clone is virtually non-existent, although not completely impossible. The window of opportunity in this system would require the addition of a genet of the opposite sex for seed production to be likely.

Changes being experienced in climate could be particularly problematic if temperature or other environmental shifts challenge the resilience of mega-clone *Tt*-IRL1. Bet-hedging in peripheral populations may be too extreme when effective population size too low. A species range of occupancy may be limited by environmental parameters such as temperature range (Brown and Eckert, 2005). Alternatively, dispersal may be the main factor limiting recruitment (Eriksson and Froborg, 1996; Kendrick et al., 2012) leading to small population size where there is a lack of propagules for recruitment. If a clone such as mega-clone *Tt*-IRL1 is lost, such as expected with climate changes, the components of this genets resilience that enable its survival beyond the maintenance state would have been exceeded. Comparison with two large populations of *Thalassia testudium* from Florida Bay, further south, indicated a relatively high genetic distance among sites. This is expected given the potential for random genetic drift and recruitment limitation into the IRL even should these areas be potential source populations for new propagules.

The most immediate concern is that environmental or biological calamities could threaten this clones existence and eliminate more than 47 km of the species range. How species will adapt to predicted changes in climate will critically influence global ecosystem dynamics, making a species range of adaptive capacity and ability to persist critical. Capacity of a species to adapt at its margin substantiates potential resilience to stressful habitats. In stressful habitats species like turtlegrass survive through continual growth of a robust clone, repeating the opportunity to bet over-and-over on seed production if, and when, possible. Populations comprised of long lived individuals could therefore make excellent barometers of environmental change as significant demographic change indicates conditions have strayed beyond the environmental continuum they have the capacity to tolerate. The unusual circumstances that have allowed us to detect the mega-clone *Tt*-IRL1 will be rarely repeated, because in populations with more than one member the clone may hide below detection thresholds in dense meadows. It may not be that these large clones are rare, but rather it is rare to detect them. Contrary to the assumption that such a clone represents a highly effective ecotype, we believe the size and longevity of this turtlegrass clone means we have found a phenotypically flexible generalist. This is important because persistence of this type reveals a species capable of maintaining habitat and ecological structure throughout the dynamic ecological changes that occur on larger time scales.

## Author Contributions

This project was a collaboration between all the authors who contributed to different components of the experimental design, data collection, analysis and interpretation, and writing. EB led the field work. EB and AC completed the laboratory analysis. EB and MW were responsible for the experimental design and analysis of the molecular data. EB, RV, and MW completed interpretation of environmental conditions and models of growth. AC produced the figures. All the authors contributed to the writing and editing of the manuscript. This work initially formed a component of EB’s Ph.D. thesis at the University of Virginia.

## Conflict of Interest Statement

The authors declare that the research was conducted in the absence of any commercial or financial relationships that could be construed as a potential conflict of interest.
