# Supplementary material for: Mega Clonality in an Aquatic Plant—A Potential Survival Strategy in a Changing Environment
Source: Front Plant Sci. 2018 Apr 6;9:435. doi: 10.3389/fpls.2018.00435 (PMC5897627; doi:10.3389/fpls.2018.00435)
Supplement: Supplementary file 1 [file Table_1.DOCX]

Supplementary Materials

*Mega clonality in an aquatic plant—a potential survival strategy in a changing environment*

Eric Bricker^1^, Ainsley Calladine^2,4^, Robert Virnstein^3^, Michelle Waycott^4,2^*

^1^ University of Virginia, Department of Environmental Sciences, Virginia USA

^2^ State Herbarium of South Australia, Department for Environment and Water, South Australia

^3^ Seagrass Ecosystems Analysts, Florida USA

^4^ School of Biological Sciences, The University of Adelaide, South Australia, Australia

*correspondence email: michelle.waycott@adelaide.edu.au

**Supplemental Table 1.** Site-specific sampling, and associated number of genets identified among these samples, across all Indian River Lagoon (IRL) collection locations.

| **Site** | **Ramets collected** | **Genets identified in each site** | **Percentage of sample genotyped as Mega Clone** |
| --- | --- | --- | --- |
| Oslo Road – Site 1 | 75 | 1 | 100% |
| Harbor Branch – Site 2 | 75 | 1 | 100% |
| Green House – Site 3 | 75 | 1 | 100% |
| Commercial Boat Dock –  Site 4 (note - landward side of Ft. Pierce Inlet) | 50 | 4 | 93% |
| Ft. Pierce Inlet – Site 5 | 8 | 1 | 100% |
| Jensen Beach-Site 6 | 25 | 1 | 100% |
| Jensen Beach 2 – Site 7 | 17 | 1 | 100% |
| St Lucie Inlet – Site 8 | 57 | 23 (18 ramets genotyped as Mega Clone, some other genets also detected across multiple ramets) | 32% |
| **Total** | **382** | **26 (340 ramets genotyped as Mega Clone)** | **89%** |
