# Supplementary material for: Mega Clonality in an Aquatic Plant—A Potential Survival Strategy in a Changing Environment
Source: Front Plant Sci. 2018 Apr 6;9:435. doi: 10.3389/fpls.2018.00435 (PMC5897627; doi:10.3389/fpls.2018.00435)
Supplement: Supplementary file 2 [file Table_2.DOCX]

Supplementary Materials

*Mega clonality in an aquatic plant—a potential survival strategy in a changing environment*

Eric Bricker^1^, Ainsley Calladine^2,4^, Robert Virnstein^3^, Michelle Waycott^4,2^*

^1^ University of Virginia, Department of Environmental Sciences, Virginia USA

^2^ State Herbarium of South Australia, Department for Environment and Water, South Australia

^3^ Seagrass Ecosystems Analysts, Florida USA

^4^ School of Biological Sciences, The University of Adelaide, South Australia, Australia

*correspondence email: michelle.waycott@adelaide.edu.au

**Supplemental Table 2.** Pairwise genetic distances for all *Thalassia testudinum* (*Tt*-IRL) genets detected at the St Lucie collection area. The Mega clone detected across 47 km of the Indian River Lagoon is *Tt*-IRL1 (**in bold**) and is included for reference. Genetic distance was calculated using GenAlEx 6.5.b3^2,3^.

| *Genet number* | *2* | *3* | *4* | *5* | *6* | *7* | *8* | *9* | *10* | *11* | *12* | *13* | *14* | *15* | *16* | *17* | *18* | *19* | *20* | *21* | *22* | *23* |
| --- | --- | --- | --- | --- | --- | --- | --- | --- | --- | --- | --- | --- | --- | --- | --- | --- | --- | --- | --- | --- | --- | --- |
| ***Tt*-IRL1** | **8** | **9** | **9** | **13** | **12** | **9** | **9** | **10** | **10** | **8** | **9** | **13** | **9** | **11** | **9** | **10** | **9** | **8** | **8** | **8** | **9** | **9** |
|  |  |  |  |  |  |  |  |  |  |  |  |  |  |  |  |  |  |  |  |  |  |  |
| *Tt*-IRL 2 | - |  |  |  |  |  |  |  |  |  |  |  |  |  |  |  |  |  |  |  |  |  |
| *Tt*-IRL 3 | 4 | - |  |  |  |  |  |  |  |  |  |  |  |  |  |  |  |  |  |  |  |  |
| *Tt*-IRL 4 | 1 | 5 | - |  |  |  |  |  |  |  |  |  |  |  |  |  |  |  |  |  |  |  |
| *Tt*-IRL 5 | 4 | 7 | 5 | - |  |  |  |  |  |  |  |  |  |  |  |  |  |  |  |  |  |  |
| *Tt*-IRL 6 | 5 | 7 | 6 | 3 | - |  |  |  |  |  |  |  |  |  |  |  |  |  |  |  |  |  |
| *Tt*-IRL 7 | 6 | 2 | 7 | 9 | 8 | - |  |  |  |  |  |  |  |  |  |  |  |  |  |  |  |  |
| *Tt*-IRL 8 | 5 | 1 | 6 | 8 | 7 | 1 | - |  |  |  |  |  |  |  |  |  |  |  |  |  |  |  |
| *Tt*-IRL 9 | 5 | 2 | 6 | 7 | 6 | 2 | 1 | - |  |  |  |  |  |  |  |  |  |  |  |  |  |  |
| *Tt*-IRL10 | 6 | 3 | 7 | 8 | 7 | 2 | 2 | 1 | - |  |  |  |  |  |  |  |  |  |  |  |  |  |
| *Tt*-IRL11 | 1 | 5 | 2 | 5 | 5 | 5 | 4 | 4 | 5 | - |  |  |  |  |  |  |  |  |  |  |  |  |
| *Tt*-IRL12 | 3 | 6 | 4 | 7 | 7 | 6 | 5 | 5 | 6 | 2 | - |  |  |  |  |  |  |  |  |  |  |  |
| *Tt*-IRL13 | 6 | 7 | 7 | 2 | 1 | 8 | 7 | 6 | 7 | 6 | 8 | - |  |  |  |  |  |  |  |  |  |  |
| *Tt*-IRL14 | 2 | 4 | 3 | 3 | 3 | 5 | 4 | 4 | 5 | 2 | 5 | 3 | - |  |  |  |  |  |  |  |  |  |
| *Tt*-IRL15 | 6 | 7 | 7 | 5 | 3 | 6 | 7 | 6 | 6 | 6 | 8 | 3 | 4 | - |  |  |  |  |  |  |  |  |
| *Tt*-IRL16 | 3 | 5 | 4 | 6 | 6 | 6 | 5 | 5 | 6 | 3 | 2 | 7 | 4 | 7 | - |  |  |  |  |  |  |  |
| *Tt*-IRL17 | 5 | 6 | 6 | 6 | 6 | 6 | 5 | 5 | 6 | 4 | 4 | 6 | 5 | 7 | 5 | - |  |  |  |  |  |  |
| *Tt*-IRL18 | 4 | 5 | 5 | 5 | 5 | 6 | 5 | 5 | 6 | 4 | 4 | 5 | 4 | 6 | 3 | 2 | - |  |  |  |  |  |
| *Tt*-IRL19 | 5 | 3 | 6 | 8 | 6 | 3 | 3 | 4 | 4 | 5 | 8 | 6 | 3 | 5 | 7 | 7 | 6 | - |  |  |  |  |
| *Tt*-IRL20 | 4 | 2 | 5 | 7 | 5 | 3 | 2 | 3 | 4 | 4 | 7 | 5 | 2 | 5 | 6 | 6 | 5 | 1 | - |  |  |  |
| *Tt*-IRL21 | 5 | 3 | 6 | 8 | 6 | 3 | 3 | 4 | 4 | 5 | 8 | 6 | 3 | 5 | 7 | 7 | 6 | 1 | 1 | - |  |  |
| *Tt*-IRL22 | 4 | 3 | 5 | 6 | 4 | 4 | 3 | 2 | 3 | 4 | 7 | 4 | 2 | 4 | 6 | 6 | 5 | 2 | 1 | 2 | - |  |
| *Tt*-IRL23 | 3 | 4 | 4 | 4 | 4 | 5 | 4 | 4 | 5 | 3 | 3 | 4 | 3 | 5 | 2 | 3 | 1 | 5 | 4 | 5 | 4 | - |

**References for supplemental material**

1 van Dijk, J. K., Waycott, M., van Tussenbroek, B. I. & Ouborg, J. Polymorphic microsatellite markers for the Caribbean seagrass *Thalassia testudinum Banks ex Konig*. *Mol Ecol Notes* **7**, 89-91, doi:10.1111/J.1471-8286.2006.01539.X (2007).

2 Nei, M. Estimation of average heterozygosity and genetic distance from a small number of individuals. *Genetics* **89**, 583-590 (1978).

3 Peakall, R. & Smouse, P. E. GENALEX 6: genetic analysis in Excel. population genetic software for teaching and research. *Mol Ecol Notes* **6**, 288-295, doi:Doi 10.1111/J.1471-8286.2005.01155.X (2006).
